# Interleukin 11 therapy causes acute left ventricular dysfunction

**DOI:** 10.1093/cvr/cvae224

**Published:** 2024-10-09

**Authors:** Mark Sweeney, Katie O’Fee, Chelsie Villanueva-Hayes, Ekhlas Rahman, Michael Lee, Chung Nga Tam, Eneko Pascual-Navarro, Henrike Maatz, Eric L Lindberg, Konstantinos Vanezis, Chrishan J Ramachandra, Ivan Andrew, Emma R Jennings, Wei-Wen Lim, Anissa A Widjaja, David Carling, Derek J Hausenloy, Norbert Hübner, Paul J R Barton, Stuart A Cook

**Affiliations:** MRC-Laboratory of Medical Sciences, Hammersmith Hospital Campus, London W12 0NN, UK; Institute of Clinical Sciences, Faculty of Medicine, Imperial College London, London W12 0NN, UK; MRC-Laboratory of Medical Sciences, Hammersmith Hospital Campus, London W12 0NN, UK; Institute of Clinical Sciences, Faculty of Medicine, Imperial College London, London W12 0NN, UK; MRC-Laboratory of Medical Sciences, Hammersmith Hospital Campus, London W12 0NN, UK; Institute of Clinical Sciences, Faculty of Medicine, Imperial College London, London W12 0NN, UK; MRC-Laboratory of Medical Sciences, Hammersmith Hospital Campus, London W12 0NN, UK; Institute of Clinical Sciences, Faculty of Medicine, Imperial College London, London W12 0NN, UK; National Heart and Lung Institute, Imperial College London, London W12 0NN, UK; MRC-Laboratory of Medical Sciences, Hammersmith Hospital Campus, London W12 0NN, UK; MRC-Laboratory of Medical Sciences, Hammersmith Hospital Campus, London W12 0NN, UK; Institute of Clinical Sciences, Faculty of Medicine, Imperial College London, London W12 0NN, UK; Cardiovascular and Metabolic Sciences, Max Delbrück Center for Molecular Medicine in the Helmholtz Association (MDC), 13092 Berlin, Germany; DZHK (German Centre for Cardiovascular Research), Partner Site Berlin, Berlin, Germany; Cardiovascular and Metabolic Sciences, Max Delbrück Center for Molecular Medicine in the Helmholtz Association (MDC), 13092 Berlin, Germany; MRC-Laboratory of Medical Sciences, Hammersmith Hospital Campus, London W12 0NN, UK; Institute of Clinical Sciences, Faculty of Medicine, Imperial College London, London W12 0NN, UK; National Heart and Lung Institute, Imperial College London, London W12 0NN, UK; National Heart Research Institute Singapore, National Heart Centre Singapore, Singapore 169609, Singapore; Cardiovascular and Metabolic Disorders Program, Duke-National University of Singapore Medical School, Singapore 169857, Singapore; MRC-Laboratory of Medical Sciences, Hammersmith Hospital Campus, London W12 0NN, UK; Institute of Clinical Sciences, Faculty of Medicine, Imperial College London, London W12 0NN, UK; MRC-Laboratory of Medical Sciences, Hammersmith Hospital Campus, London W12 0NN, UK; Institute of Clinical Sciences, Faculty of Medicine, Imperial College London, London W12 0NN, UK; National Heart Research Institute Singapore, National Heart Centre Singapore, Singapore 169609, Singapore; Cardiovascular and Metabolic Disorders Program, Duke-National University of Singapore Medical School, Singapore 169857, Singapore; Cardiovascular and Metabolic Disorders Program, Duke-National University of Singapore Medical School, Singapore 169857, Singapore; MRC-Laboratory of Medical Sciences, Hammersmith Hospital Campus, London W12 0NN, UK; Institute of Clinical Sciences, Faculty of Medicine, Imperial College London, London W12 0NN, UK; National Heart Research Institute Singapore, National Heart Centre Singapore, Singapore 169609, Singapore; Cardiovascular and Metabolic Disorders Program, Duke-National University of Singapore Medical School, Singapore 169857, Singapore; Yong Loo Lin School of Medicine, National University of Singapore, Singapore 117597, Singapore; The Hatter Cardiovascular Institute, University College London, London WC1E 6HX, UK; Cardiovascular and Metabolic Sciences, Max Delbrück Center for Molecular Medicine in the Helmholtz Association (MDC), 13092 Berlin, Germany; DZHK (German Centre for Cardiovascular Research), Partner Site Berlin, Berlin, Germany; Charite, Universitätsmedizin Berlin, 10117 Berlin, Germany; MRC-Laboratory of Medical Sciences, Hammersmith Hospital Campus, London W12 0NN, UK; Institute of Clinical Sciences, Faculty of Medicine, Imperial College London, London W12 0NN, UK; National Heart and Lung Institute, Imperial College London, London W12 0NN, UK; Royal Brompton and Harefield Hospitals, Guy’s and St. Thomas’ NHS Foundation Trust, London SW3 6NP, UK; MRC-Laboratory of Medical Sciences, Hammersmith Hospital Campus, London W12 0NN, UK; Institute of Clinical Sciences, Faculty of Medicine, Imperial College London, London W12 0NN, UK; National Heart Research Institute Singapore, National Heart Centre Singapore, Singapore 169609, Singapore; Cardiovascular and Metabolic Disorders Program, Duke-National University of Singapore Medical School, Singapore 169857, Singapore

**Keywords:** Interleukin 11, Heart failure, Fibrosis, Inflammation, JAK/STAT, Cardiotoxicity

## Abstract

**Aims:**

Interleukin 11 (IL11) was initially thought important for platelet production, which led to recombinant IL11 being developed as a drug to treat thrombocytopenia. IL11 was later found to be redundant for haematopoiesis, and its use in patients is associated with unexplained and severe cardiac side effects. Here, we aim to identify, for the first time, direct cardiomyocyte toxicities associated with IL11, which was previously believed cardioprotective.

**Methods and results:**

We injected recombinant mouse lL11 (rmIL11) into mice and studied its molecular effects in the heart using immunoblotting, qRT-PCR, bulk RNA-seq, single nuclei RNA-seq (snRNA-seq), and assay for transposase-accessible chromatin with sequencing (ATAC-seq). The physiological impact of IL11 was assessed by echocardiography *in vivo* and using cardiomyocyte contractility assays *in vitro*. To determine the activity of IL11 specifically in cardiomyocytes, we made two cardiomyocyte-specific *Il11ra1* knockout (CMKO) mouse models using either AAV9-mediated and *Tnnt2*-restricted (vCMKO) or *Myh6* (m6CMKO) Cre expression and an *Il11ra1* floxed mouse strain. In pharmacologic studies, we studied the effects of JAK/STAT inhibition on rmIL11-induced cardiac toxicities. Injection of rmIL11 caused acute and dose-dependent impairment of left ventricular ejection fraction (saline: 62.4% ± 1.9; rmIL11: 32.6% ± 2.9, *P* < 0.001, *n* = 5). Following rmIL11 injection, myocardial STAT3 and JNK phosphorylation were increased and bulk RNA-seq revealed up-regulation of pro-inflammatory pathways (TNFα, NFκB, and JAK/STAT) and perturbed calcium handling. snRNA-seq showed rmIL11-induced expression of stress factors (*Ankrd1*, *Ankrd23*, *Xirp2*), activator protein-1 (AP-1) transcription factor genes, and *Nppb* in the cardiomyocyte compartment. Following rmIL11 injection, ATAC-seq identified the *Ankrd1* and *Nppb* genes and loci enriched for stress-responsive, AP-1 transcription factor binding sites. Cardiomyocyte-specific effects were examined in vCMKO and m6CMKO mice, which were both protected from rmIL11-induced left ventricular impairment and molecular pathobiologies. In mechanistic studies, inhibition of JAK/STAT signalling with either ruxolitinib or tofacitinib prevented rmIL11-induced cardiac dysfunction.

**Conclusions:**

Injection of IL11 directly activates IL11RA/JAK/STAT3 in cardiomyocytes to cause acute heart failure. Our data overturn the earlier assumption that IL11 is cardioprotective and explain the serious cardiac side effects associated with IL11 therapy.

## Graphical Abstract

**Graphical Abstract:**
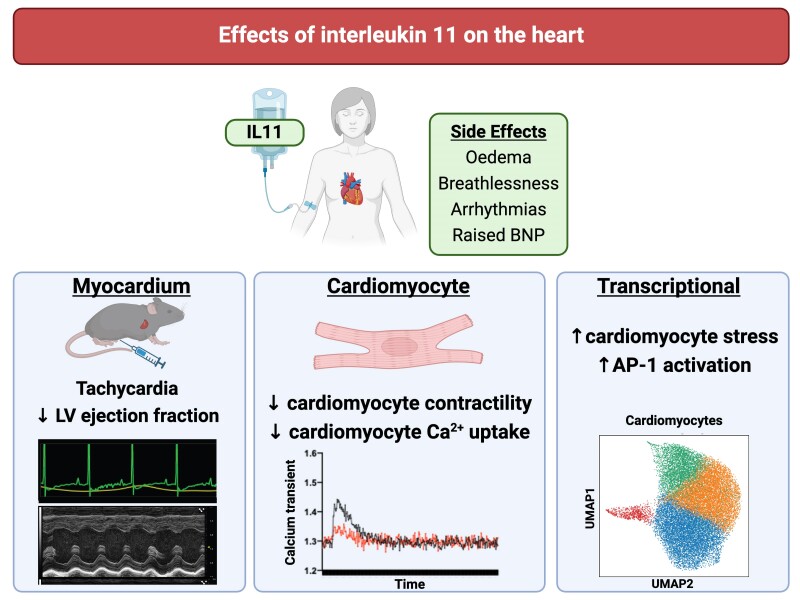

**Time of primary review: 18 days**

**See the editorial comment for this article ‘Inflammation and heart failure: are we facing a ‘hedgehog’s dilemma'?’, by S. Ministrini and G.G. Camici, https://doi.org/10.1093/cvr/cvae246.**

## 1. Introduction

Interleukin 11 (IL11) is an elusive member of the interleukin 6 (IL6) family of cytokines, which collectively signal via the gp130 co-receptor. Following its identification in 1990,^1^ recombinant human IL11 (rhIL11) was found to increase megakaryocyte activity and peripheral platelet counts in mice.^2^ Soon after, IL11 was developed as a therapeutic (Oprelvekin; Neumega) to increase platelet counts in patients with chemotherapy-induced thrombocytopenia, received FDA approval for this indication in 1998, and is still used to this day.^3,4^ In recent years, longer-acting formulations of rhIL11 have been tested in pre-clinical studies and new clinical trials of PEGylated rhIL11 in patients are anticipated.^5^

RhIL11 was also trialled to increase platelet counts in patients with von Willebrand factor deficiency, myelodysplastic syndrome, cirrhosis and sepsis, and tested as a putative cytoprotective agent in numerous other conditions, including myocardial infarction^6^ (*Table 1* and Supplementary material online, *Table S1*). However, it became apparent that IL11 is not required for basal or compensatory red blood cell or platelet production in mice or humans: IL11 is in fact redundant for haematopoiesis.^7,8^ Thus, the effects of injection of high dose rhIL11 on platelets appear non-physiological and possibly reflect non-specific gp130 activity.^9,10^

**Table 1:** Human clinical trials registered with clinicaltrials.gov using recombinant human interleukin 11

NCT number	Title	Start date	*n*	Status	Phase
Thrombocytopenia					
NCT03823079	Comparison of Interleukin-11 and rhTPO for Recurrent Colorectal Cancer Patients With Thrombocytopenia	Feb-19	50	Unknown status	2
NCT01663441	A Phase IIIa Study of Genetically Modified Recombinant Human Interleukin-11	Mar-15	62	Completed	3
NCT02314273	Effect of rhIL-11 in Patients With Thrombocytopenia for Childhood Acute Lymphocytic Leukaemia	Sep-11	120	Completed	4
NCT00886743	Study Evaluating The Effects Of Oprelvekin On Cardiac Repolarization In Subjects With Chemotherapy Induced Thrombocytopenia	Sep-09	19	Terminated	2
NCT00493181	Interleukin 11, Thrombocytopenia, Imatinib in Chronic Myelogenous Leukemia Patients	Oct-05	8	Completed	2
Coagulopathy					
NCT00994929	Efficacy and Safety of IL-11 in DDAVP Unresponsive	Jan-10	9	Completed	2
NCT00524225	IL-11 in Adults With Von Willebrand Disease Undergoing Surgery	Feb-08	3	Terminated	2
NCT00524342	IL-11 in Women With Von Willebrand Disease and Refractory Menorrhagia	Jan-08	7	Completed	2
NCT00151125	Phase II Study of IL-11 (Neumega) in Von Willebrand Disease	Jul-04	12	Completed	2
Inflammatory bowel disease					
NCT00038922	Study Evaluating rhIL-11 in Left-Sided Ulcerative Colitis	Jun-02	–	Terminated	1
NCT00040521	Study Evaluating rhIL-11 in Active Crohn’s Disease	Apr-02	–	Completed	2
Other					
NCT00012298	Radiolabeled Monoclonal Antibody Plus Rituximab With and Without Filgrastim and Interleukin-11 in Treating Patients With Relapsed or Refractory Non-Hodgkin’s Lymphoma	Apr-01	81	Terminated	1/2
NCT03720340	Interleukin-11 Can Prevent and Treat of Radioactive Oral Mucitis	Oct-18	300	Unknown status	3

Unfortunately, injection of rhIL11 into patients has severe and hitherto unexplained cardiac side effects. Up to 20% of patients given rhIL11 (50 µg/kg) develop atrial arrhythmias, a high proportion of individuals develop heart failure, and rare cases of ventricular arrhythmias and sudden death are reported.^11,12^ Furthermore, serum natriuretic peptide levels become acutely and transiently elevated in patients receiving IL11 therapy, with B-natriuretic peptide levels sometimes exceeding those diagnostic of heart failure.

While IL11 was previously thought to be cytoprotective, anti-inflammatory, and anti-fibrotic in the heart^13–15^ and other organs, recent studies by ourselves and others have challenged this premise.^16–18^ Indeed, experiments over the last five years have questioned the earlier literature and IL11 is increasingly viewed as pro-inflammatory and pro-fibrotic. Given this large shift in our understanding of IL11 and the fact that cardiomyocytes (CMs) robustly express IL11 receptors (IL11RA),^15,19,20^ we devised experiments to determine whether IL11 is toxic to CMs and if this could explain cardiac side effects associated with IL11 therapy in patients.

## 2. Methods

Detailed information on experimental methods of RNA and DNA analysis and CM isolation is provided in the Supplementary material.

### 2.1 Animal studies

All mouse studies were conducted according to the Animals (Scientific Procedures) Act 1986 Amendment Regulations 2012 and approved by the Animal Welfare Ethical Review Body at Imperial College London. Animal experiments were carried out under UK Home Office Project License P108022D1 (September 2019). Wild type (WT) mice on a C57BL/6J background were purchased from Charles River (Cat#632). They were bred in a dedicated breeding facility and housed in a single room of the experimental animal facility with a 12 h light–dark cycle and provided food and water *ad libitum*. Mice were euthanized by cervical dislocation and decapitation prior to removal of tissue for analysis.

The *Il11ra1* floxed mouse (C57BL/6-Il11ra1^em1Cook^/J, Jax:034465) has exons 4–7 of the *Il11ra1* gene flanked by loxP sites as has been described previously.^21^ In the presence of Cre-recombinase excision of exon 4–7 results in a non-functional IL11 receptor.

Male myosin heavy chain 6 Cre (*Myh6*-Cre) mice [B6.FVB-Tg(Myh6-cre)2182Mds/J, Jax:011038] were purchased from Jax (Bar Harbor, Maine, USA) as heterozygotes. These mice were crossed with homozygous *Il11ra1* floxed females. In the second generation, mice from generation one, heterozygous for the *Il11ra1* flox allele and heterozygous for the Cre, were crossed with *Il11ra1* flox homozygotes to produce litter-mate experimental and control animals.

Recombinant mouse interleukin-11 (rmIL11) (Z03052, Genscript, Oxford, UK) was dissolved in phosphate buffered saline (PBS) (14190144, ThermoFisher, MA, USA), and injected intraperitoneally (ip) at a dose of 200 µg/kg unless otherwise stated. Control mice received an equivalent volume of saline (2 µL/kg). Recombinant mouse interleukin-6 (Z02767, Genscript) was dissolved in PBS and injected ip at a dose of 200 µg/kg.

### 2.2 Genotyping

Genotype was confirmed with ear-notch DNA samples. DNA was extracted using a sodium hydroxide digestion buffer, then neutralized with 1 M Tris-HCl pH 8. *Il11ra1* flox genotype was confirmed with a single polymerase chain reaction (PCR) reaction yielding a PCR product at 163 bp for the wild type allele or 197 bp in the transgenic allele. *Myh6*-Cre mice were genotyped using two reactions for either the transgenic gene product of 295 bp (or wild type gene product of 300 bp) along with an internal positive control (200 bp). Primers used in these reactions are detailed in Supplementary material online, *Table S2*.

### 2.3 Viral vector

The viral vector used in this study, AAV9-cTNT-EGFP-T2A-iCre-WPRE (VB5413), was purchased from Vector Biolabs (Malvern, PA, USA). A codon optimized Cre was delivered using an adeno-association virus type 9 (AAV9) capsid and under the control of the *Tnnt2* promoter. This was linked to an enhanced green fluorescent protein (EGFP) reporter with a 2a self-cleaving linker. A total of 1 × 10^12^ genome copies or an equivalent volume of saline were injected into the tail veins of 8–9-week-old homozygous male *Il11ra1* flox mice and from this point, mice were housed separately from saline-injected controls for 4 weeks prior to further experiments.

### 2.4 Echocardiography

Echocardiography was performed under light isoflurane anaesthesia using a Vevo3100 imaging system and MX550D linear transducer (Fujifilm Visualsonic Inc., ON, Canada). Anaesthesia was induced with 4% isoflurane for 1 min and maintained with 1–2% isoflurane. Mice were allowed to equilibrate to the anaesthetic after induction for 9 min before imaging was started. Heart rate measurement from single-lead electrocardiogram (ECG) recordings was taken at the completion of the equilibration period. Measurements of ventricular ejection fraction (LVEF) were measured from m-mode images taken in the parasternal short axis (PSAX) view at mid-ventricular level and averaged across 3 heartbeats.

### 2.5 qPCR

The tissue was washed in ice-cold PBS and snap-frozen in liquid nitrogen. Total RNA was extracted using TRIzol (15596026, Invitrogen, MA, USA) in RNeasy columns (74106, Qiagen, MD, USA). cDNA was synthesized using Superscript Vilo Mastermix (11755050, Invitrogen). Gene expression analysis was performed using quantitative polymerase chain reaction (qPCR) with TaqMan gene expression assay in duplicate over 40 cycles; *Il11ra1*: custom TaqMan assay (Supplementary material online, *Table S3*), *Nppb*: Mm01255770_g1, *Rrad*: Mm00451053_m1, *Fosl2* Mm00484442_m1. Gene expression data were normalized to *Gapdh* expression (Mm99999915_g1), and fold change compared to control samples was calculated using 2^−ΔΔCt^ method.

### 2.6 RNA-seq

Eight-week-old male C57BL/6J mice were injected with rmIL11 (200 µg/kg) or an equivalent volume of saline (2 µL/kg). The left ventricle (LV) was excised and flash frozen 1, 3, or 6 h after injection. Libraries were sequenced on a NextSeq 2000 to generate a minimum of 20 million paired end 60 bp reads per sample.

### 2.7 Single nuclei RNA-seq

Single nuclei sequencing was performed on flash-frozen LV tissue that was extracted from 8-week-old male C57BL/6J mice 3 h after injection with rmIL11 or saline. The tissue was processed according to standard protocols as previously described.^22,23^ Nuclei were purified by fluorescent activated cell sorting, and libraries were sequenced using HiSeq 4000 (Illumina, CA, USA) with a minimum depth of 20 000–30 000 read pairs per nucleus.

### 2.8 ATAC-seq

Eight-week-old male C57BL/6J mice were given an ip injection with rmIL11 (200 µg/kg) or saline. The heart was excised 3 h after injection, and flash-frozen tissue was sent to Active Motif (Carlsbad, CA, USA) to perform assay for transposase-accessible chromatin with sequencing (ATAC-seq) analysis.

### 2.9 Protein analysis

Protein extraction was performed on flash-frozen tissue using ice-cold Pierce RIPA buffer (89901, ThermoFisher) supplemented with protease inhibitors (11697498001, Roche, Basel, Switzerland) and phosphatase inhibitors (4906845001, Roche). Tissue was lysed using a Qiagen Tissue Lyser II with metallic beads for 3 min at 30 Hz. Protein quantification was performed using a Pierce bicinchoninic acid assay colorimetric protein assay kit (23225, ThermoFisher). A total of 10–20 µg of protein was loaded per well and run on a 4–12% bis-Tris precast sodium-dodecyl sulfate page gel (NP0323BOX, Invitrogen). Semi-dry transfer was performed using the TransBlot Turbo transfer system (1704150, BioRad, CA, USA), and the membrane was blocked in 5% bovine serum albumin (A3803, Sigma-Aldrich, MO, USA). Primary antibodies raised against the following targets were used: signal transducer and activator of transcription 3 (STAT3) [4904S, cell signalling technology (CST), MA, USA], pSTAT3 Tyr705 (9145L, CST), extracellular signal regulated kinase (ERK) (9101S, CST), pERK (4695S, CST), c-Jun-N-terminal kinase (JNK) (sc-7345, Santa-Cruz, TX, USA), phospho-JNK (sc-6254, Santa-Cruz), green fluorescent protein (ab290, Abcam, Cambridge, UK), and glyceraldehyde-3-phosphate dehydrogenase (GAPDH) (2118L, CST). Appropriate secondary horseradish peroxidase linked antibody was incubated for 1 h with gentle agitation at room temperature and developed using chemiluminescence blotting substrate (1705061, BioRad or 34095, ThermoFisher, depending on strength of signal).

### 2.10 Cardiomyocyte extraction

CMs were extracted from the heart of 12-week-old male C57BL/6J mice. Cells were incubated in Tyrode solution (1 mM Ca, 1 mM Mg) or Tyrode solution supplemented with rmIL11 (10 ng/mL) for 2 h before recording. Cells were paced at 1 Hz (10 V, 10 ms pulse width). Cell recordings were made using the Cytocypher high-throughput microscope (Cytocypher BV, Netherlands), and the automated cell finding system was used to identify and take recordings from 20 individual cells per heart per experimental condition. Calcium recordings were performed by incubating CMs with Fura 2AM dye (1 μM) for 20 min before fluorescent recordings were taken.

### 2.11 Statistics

Statistical analyses were performed in GraphPad Prism V9.5.0 unless otherwise stated. Normality testing was performed using the Shapiro–Wilk test. Hypothesis testing for single comparisons was done using an unpaired two ways Student’s *t*-test for normally distributed data or by Mann–Whitney *U* test for non-normally distributed data.

Comparisons involving male and female mice were performed using a two-way analysis of variance (ANOVA) with Sidak’s multiple comparisons testing. Changes in expression over multiple time points were analysed using a one-way ANOVA with Sidak’s multiple comparisons testing for all time points and doses. All graphs display the mean and standard error of the mean unless stated otherwise. *P*-values in RNA-seq analysis were corrected for multiple testing using the false discovery rate (FDR) approach. A *P*-value and FDR of <0.05 were considered significant.

### 2.12 Hierarchical testing of nested data

Statistical analysis of the data from high-throughput microscopy of extracted CM experiments was analysed using a hierarchical statistical approach.^24^ This approach tests for clustering within the data as may occur due to differences in the quality of myocyte preparation on different days. This avoids pseudoreplication of multiple technical replicates of a single biological replicate but also increases statistical power compared to treating each biological extraction as a single replicate. This uses a two-level random intercept model of linear regression. The analysis was performed using R studio, and the data were presented as the mean and standard deviation and effective *n* number taking the intraclass clustering into account.

### 2.13 Figures

Graphs were prepared in GraphPad Prism V9.5.0 and R studio (Version 2023.03.0) Illustrations were created with Biorender.com, and figures were arranged in Adobe Illustrator (version 23.0.4.).

## 3. Results

### 3.1 Injection of rmIL11 to mice causes acute left ventricular dysfunction

**Figure 1:**
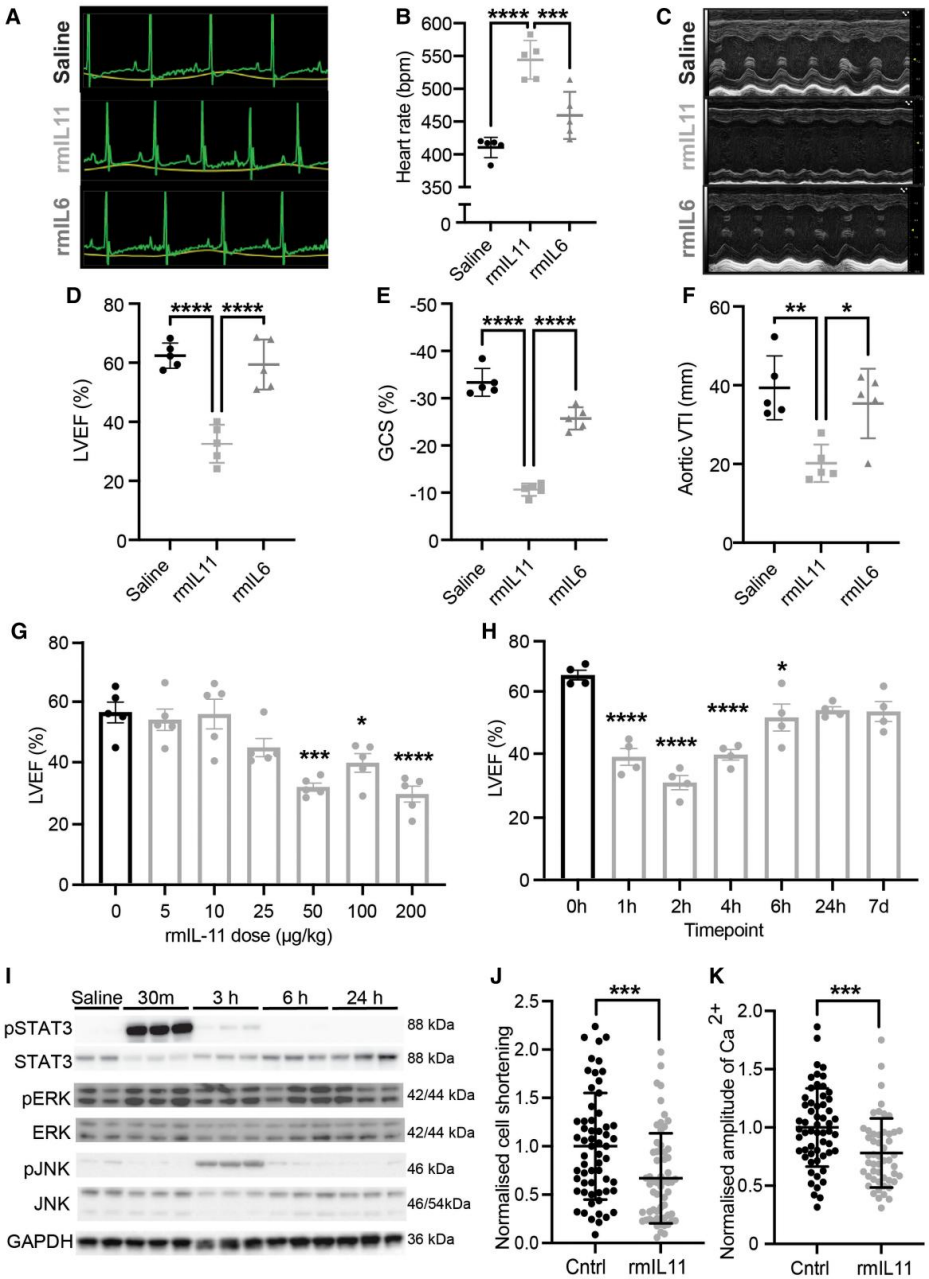
IL11 causes acute left ventricular dysfunction and impairs cardiomyocyte calcium handling. Male C57BL/6J mice were injected with rmIL11 (200 µg/kg) (▪), rmIL6 (200 µg/kg) (▴), or an equivalent volume of saline (2 µL/kg) (●). (*A*) Representative electrocardiogram traces were recorded under light anaesthesia, 2 h after intraperitoneal (ip) injection of saline, rmIL11, or rmIL6. (*B*) Quantification of heart rate (*n* = 5 per group). (*C*) Representative m-mode images from echocardiography performed 2 h after injection of saline, rmIL11, or rmIL6. (*D*) Quantification of left ventricular ejection fraction (LVEF), (*E*) global circumferential strain (GCS), and (*F*) velocity time integral at the aortic arch (VTI) in each group (*n* = 5 per group). (*G*) LVEF 2 h after ip injection of rmIL11 to male mice at 0, 5, 10, 25, 50, 100, and 200 µg/kg (*n* = 5 per dose). (*H*) LVEF at baseline, 1, 2, 4, 6, and 24 h and 7 days after ip injection of rmIL11 (200 µg/kg) (*n* = 4 per time point). (*I*) Western blot of myocardial lysates from C57BL/6J male mice 0.5, 3, 6, and 24 h after ip rmIL11 injection (200 µg/kg). Blots are probed for pSTAT3, total STAT3, pERK, total ERK, pJNK, total JNK, and GAPDH. CMs isolated from male C57BL/6J mice were treated *in vitro* for 2 h with media supplemented with rmIL11 (10 ng/mL) or non-supplemented media (Cntrl) (*n* = 3 mice, 20 cells per mouse) and assessed for (*J*) contractility (effective *n* = 9.7) and (*K*) the systolic change of intracellular calcium concentration (effective *n* = 12). Statistics: one-way ANOVA with Sidak’s multiple comparisons test. Significance denoted as *P < 0.05, **P < 0.01, ***P < 0.001, ****P < 0.0001. CM data: two-level hierarchical clustering *P*-values denoted as ***<0.001.

To model the effects of IL11 injection in clinical practice and analyse the effects on cardiac function, we injected male C57BL/6J mice intraperitoneally with rmIL11 (200 µg/kg). As compared to mice injected with saline (2 µL/kg), rmIL11-injected mice developed a sinus tachycardia [saline: 410 beats per minute (bpm) ± 6.9; rmIL11: 544 bpm ± 13, Mann–Whitney test: *P* = 0.0079, *n* = 5] (*Figure 1A* and *B*). Mice injected with rmIL11 injection had reduced LVEF (saline: 62.4% ± 1.9; rmIL11: 32.6% ± 2.9, *P* < 0.001, *n* = 5), reduced global circumferential strain (GCS) (saline: −33.4% ± 1.3; rmIL11: −10.6% ± 0.6, *P* < 0.001, *n* = 5), and reduced velocity time integral (VTI) in the aortic arch (saline: 39.4 mm ± 3.6; rmIL11: 20.2 mm ± 2.1, *P* < 0.002, *n* = 5) compared to mice injected with saline (*Figure 1C–F*) (*Table 2*). To serve as a related cytokine control, an equivalent dose (200 µg/kg) of recombinant mouse IL6 (rmIL6) was injected that had no detectable acute effects on cardiac function (*Figure 1A–F* and Supplementary material online, *Figure S1A* and *B*) (*Table 2*).

**Table 2:** Echocardiographic measures of cardiac function in saline-, rmIL11-, or rmIL6-treated mice

	Saline (*n* = 5)	rmIL11 (*n* = 5)	rmIL6 (*n* = 5)	Saline vs. rmIL11	rmIL11 vs. rmIL6
				*P*-value	*P*-value
Heart rate (bpm)	410 ± 6.9	544 ± 13	459 ± 16	0.0079*	0.004
LVEF (%)	62.4 ± 1.9	32.6 ± 2.9	59.4 ± 3.8	<0.001	<0.001
FS	27.3 ± 0.89	11.3 ± 1.1	27.9 ± 2.3	<0.001	<0.001
ESV (μL)	21.5 ± 4.4	42.6 ± 4.4	23.7 ± 2.8	0.010	0.007
EDV (μL)	55.8 ± 9.3	63.3 ± 6.1	58.1 ± 2.7	0.522	0.462
Stroke volume (μL)	34.4 ± 5.0	20.6 ± 2.6	34.4 ± 2.4	0.039	0.004
GCS (%)	−33.4 ± 1.3	−10.6 ± 0.6	−25.7 ± 1.1	<0.001	<0.001
GLS (%)	−19.8 ± 1.5	−12.3 ± 1.6	−16.5 ± 1.4	0.010	0.086
VTI (mm)	39.4 ± 3.6	20.2 ± 2.1	35.4 ± 4.0	0.002	0.010

Wild type C57BL/6J mice were injected with saline (2 μL/kg), rmIL11 (200 µg/kg), or rmIL6 (200 µg/kg), and echocardiographic measures were recorded under isoflurane anaesthesia after 2 h. Values are presented as mean ± S.E.M. Statistics: comparison between groups by one-way ANOVA with Sidak’s multiple comparisons unless otherwise indicated. Values marked with * were not normally distributed, and therefore significance was tested using Mann–Whitney *U* test. *P*-values less than 0.05 are considered significant.

bpm, beats per minute; LVEF, left ventricular ejection fraction; FS, fractional shortening; ESV, end-systolic volume; EDV, end-diastolic volume; GCS, global circumferential strain; GLS, global longitudinal strain; VTI, velocity time integral from pulse wave Doppler trace in the aortic arch.

Dosing studies revealed that the effects of rmIL11 on heart rate and LV function were dose-dependent, consistent with physiological binding to and activation of the lL11RA1 receptor. Cardiac impairment was evident at low doses and near-maximal effects were seen with a dose of 50 µg/kg, which is the dose typically given daily to patients with thrombocytopenia post-chemotherapy (*Figure 1G*). The effect of rmIL11 was rapid with a nadir in cardiac function 2 h post-injection, and recovery of LV function was seen by 24 h post-injection (*Figure 1H*).

### 3.2 IL11 causes impaired cardiomyocyte calcium handling

We next examined IL11 signalling pathways in cardiac extracts following rmIL11 injection, which revealed early and short-lived phosphorylation of signal transducer and activator of transcription 3 (p-STAT3) but no apparent ERK activation, which differs from acute signalling effects in the liver and other organs^25^ (*Figure 1I* and Supplementary material online, *Figure S1C*). Phosphorylation of JNK is a stress-related signalling pathway shown to be elevated in the mouse liver following IL11 treatment.^25^ In the myocardium, JNK was phosphorylated at the 3 h time point post-rmIL11 injection by which stage STAT3 phosphorylation was declining (*Figure 1I* and Supplementary material online, *Figure S1D*).

The effect of IL11 directly on CMs was analysed *in vitro* by treating isolated adult mouse CMs with rmIL11 for 2 h. CMs treated with rmIL11 demonstrated reduced contractility, as compared to control cells (control: 1.00 ± 0.18; rmIL11: 0.67 ± 0.15, *P* < 0.00027) (*Figure 1J*). Intracellular calcium transients revealed blunting of the peak calcium concentration during systole in the presence of rmIL11 (control: 1.00 ± 0.097; rmIL11: 0.78 ± 0.086, *P* < 0.00019) (*Figure 1K*).

### 3.3 IL11 causes cardiac inflammation

The robust and early activation of STAT3 by IL11 led us to explore transcriptional changes that might occur acutely within the myocardium in response to IL11 injection. Bulk RNA sequencing was performed on LV tissue at 1, 3, and 6 h following injection of rmIL11 and compared to controls injected with saline.

**Figure 2:**
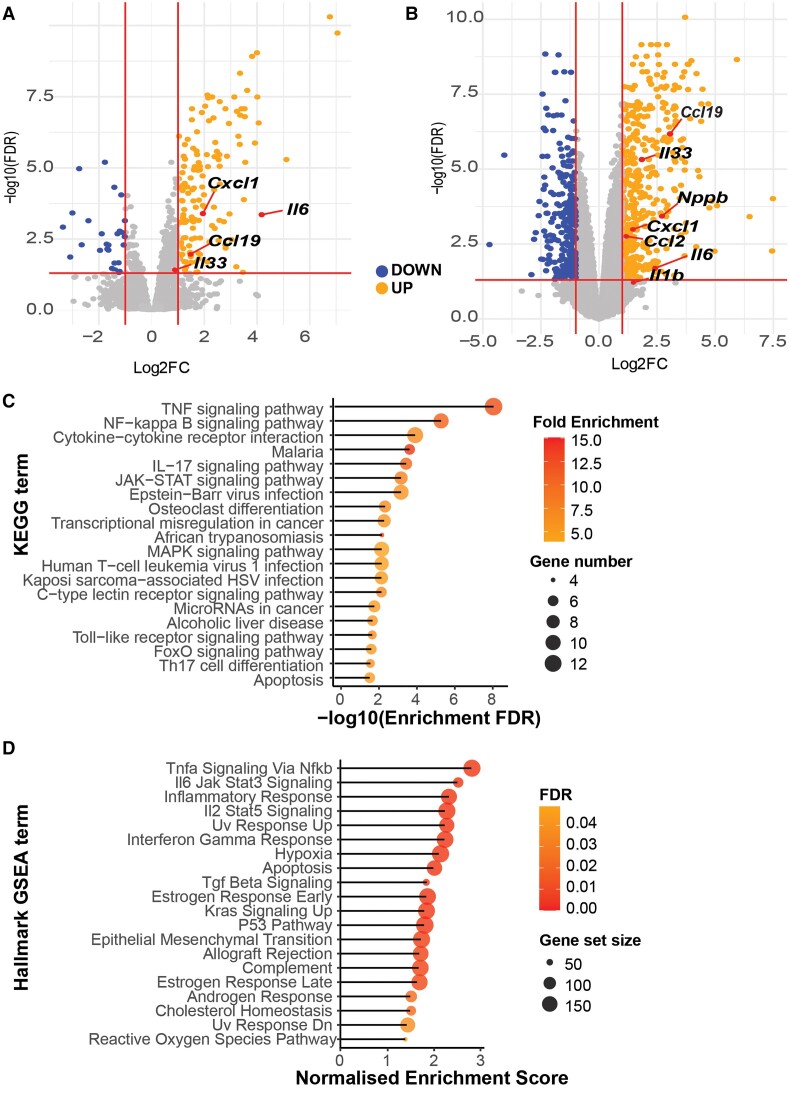
Transcriptional changes in the myocardium following rmIL11 injection. Volcano plot of all detected genes (*A*) 1 h (*n* = 3) and (*B*) 3 h (*n* = 4) after intraperitoneal injection of rmIL11 at 200 µg/kg. Vertical red lines are drawn at Log2FC of 1 and −1 and horizontal red lines at FDR of 0.05. (*C*) Chart of most significantly enriched KEGG terms from at 1 h post-injection of rmIL11 ranked by FDR. (*D*) Gene set enrichment analysis of the most highly enriched Hallmark gene sets from RNA-seq data at 1 h after injection of rmIL11 ranked by normalized enrichment score.

Extensive and significant transcription changes were apparent at all time points (1 h, up: 145, down: 27; 3 h, up: 450, down: 303; 6 h: up: 268, down: 169; Log_2_FC ± 1, FDR < 0.05). Genes differentially regulated included early up-regulation of acute inflammatory genes (*Il6*, *Il1b*, and *Il33*), chemotactic factors such as *Ccl2* and *Cxcl1*, and CM stress markers (*Nppb*, *Cnn2*, *Ankrd1*) (*Figure 2A* and *B*). Kyoto Encyclopedia of Genes and Genomes (KEGG) analysis of the differentially expressed genes at the 1 h time point revealed the tumour necrosis factor α (TNFα), NFκB and Janus kinase (JAK)/STAT signalling were among the most significantly enriched terms (*Figure 2C* and Supplementary material online, *Figure S2A* and *C*). A similar group of inflammatory terms were highlighted by Hallmark Gene Set Enrichment Analysis including TNFα signalling via NFκB, IL6 JAK/STAT, and interferon-gamma signalling (*Figure 2D* and Supplementary material online, *Figure S2B* and *D*). These transcriptional changes show that IL11 drives an acute pro-inflammatory response in the heart that is associated with impaired systolic function.

### 3.4 Single nuclear sequencing reveals a cardiomyocyte stress signature

**Figure 3:**
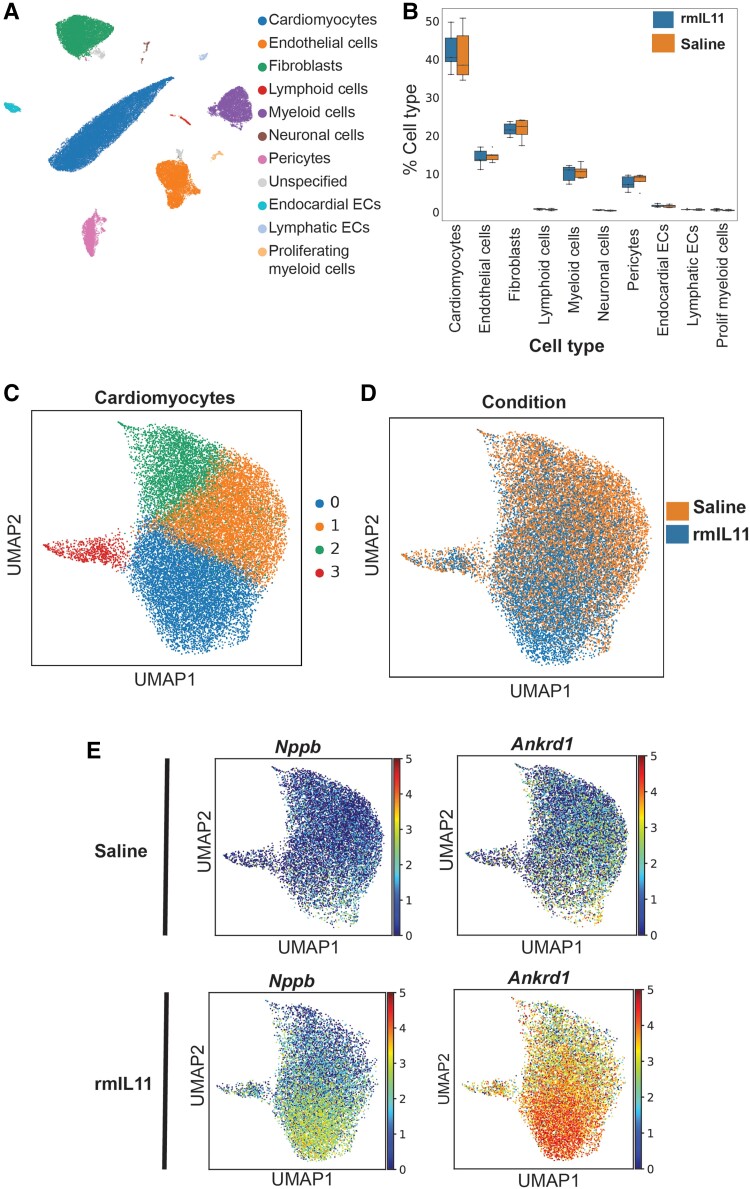
Single nuclear RNA sequencing reveals an IL11-induced cardiomyocyte stress signature. (*A*) Uniform Manifold Approximation and Projection (UMAP) embedding of all cell types from the left ventricle of male C57BL/6J mice 3 h after intraperitoneal injection of rmIL11 (200 µg/kg) or an equivalent volume of saline (*n* = 5). (*B*) Comparison of cellular composition of the left ventricle in rmIL11-treated mice compared to saline-treated mice. (*C*) UMAP embedding of cardiomyocyte fraction. Four distinct clusters are identified based on gene expression. (*D*) UMAP embedding of cardiomyocytes annotated with the treatment group. (*E*) UMAP embedding of cardiomyocyte fraction of saline- or rmIL11-treated cardiomyocytes annotated with relative expression of *Nppb* and *Ankrd1*. EC, endothelial cells.

To examine cell-specific transcriptional responses and define any potential changes in cell populations, we performed single nucleus RNA sequencing (snRNA-seq) experiments on hearts 3 h post-rmIL11 injection (*Figure 3A*, Supplementary material online, *Figures S3A*–*C* and *S4A*, and Supplementary material online, *Table S4*). This revealed no significant change in cell populations overall, excluding immune cell infiltration at this early time point (*Figure 3B*) although chronic IL11 expression is known to cause immune cell infiltration.^18^

On closer analysis of CMs, this cell type segregated into four states with rmIL11-treated CM predominantly clustered in state 0 (*Figure 3C* and *D*). This state is defined by the expression of a number of cardiomyocyte stress factors including *Ankrd1*, *Ankrd23*, and *Xirp2* (*Figure 3E* and Supplementary material online, *Figure S4B*). *Ankrd1* and *Ankrd23* are stress-inducible ankyrin repeat proteins that are elevated in dilated cardiomyopathies.^26,27^*Xirp2* encodes cardiomyopathy-associated protein 3 and is up-regulated in CMs in response to stress.^28,29^ Expression of *Nppb*, a canonical heart failure gene, was similarly elevated (*Figure 3E*). Overall, the most enriched pathway from KEGG analysis of CM-specific differentially expressed genes, irrespective of state, was ‘Ribosome’ with 93 out of 130 genes significantly up-regulated (fold enrichment: 4.5, FDR: 2.3e^−46^), perhaps related to the large effects of IL11 on protein translation within CMs to cellular stress (Supplementary material online, *Figure S5*).^30^

### 3.5 ATAC-Seq highlights AP-1 family genes

To better understand the molecular changes induced by IL11 in the heart, we performed an assay for transposase-accessible chromatin using sequencing (ATAC-seq) analysis. This methodology identifies regions of the genome undergoing epigenetic variation to make transcription factor binding sites more or less accessible.

**Figure 4:**
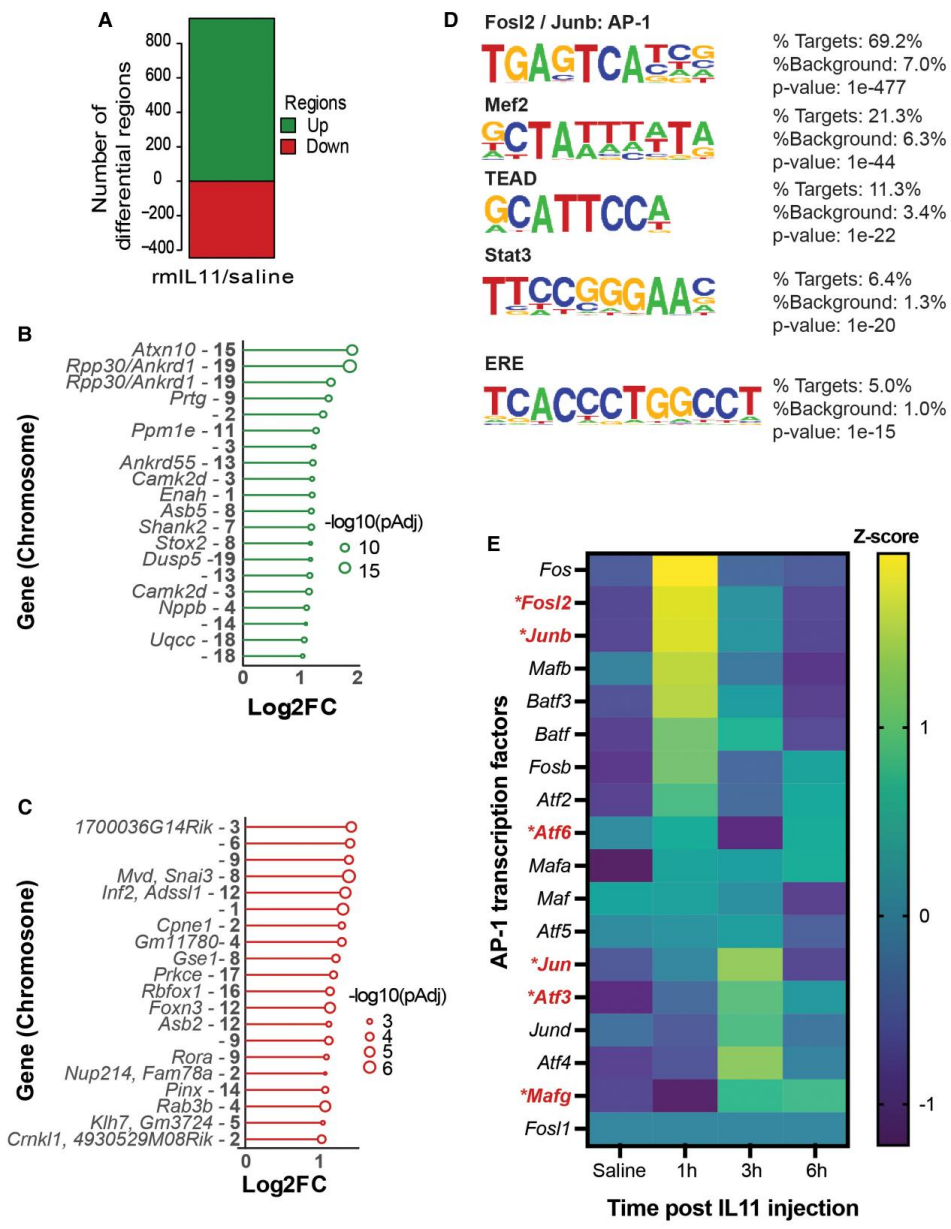
ATAC-Seq reveals a stress signature that occurs acutely in the myocardium after rmIL11 injection. (*A*) Number of positively and negatively enriched genomic regions identified by ATAC-Seq analysis of the myocardium 3 h after injection of rmIL11 (*n* = 4). (*B*) Top 20 most strongly enriched DNA regions in ATAC-seq analysis and adjacent genes, when present (gene–chromosome). (*C*) Top 20 most strongly negatively enriched DNA regions in ATAC-seq analysis and adjacent genes (gene–chromosome). (*D*) *De novo* Homer motif analysis of ATAC-seq data most highly enriched motifs in myocardial samples. (*E*) Heatmap of AP-1 transcription factor family members from bulk RNA sequencing data of myocardium at baseline, 1, 3, and 6 h after rmIL11 injection. Genes differentially expressed in cardiomyocytes in single nuclear RNA sequencing data are marked with * and highlighted in red.

Following IL11 administration, there were a large number of loci with variation in DNA accessibility (increased: 945; reduced: 445, shrunkenLog2FC: ±0.3, *P*adj < 0.1) (*Figure 4A* and Supplementary material online, *Table S5*). The top 20 most differentially enriched regions (*Figure 4B* and *C*) include areas adjacent to *Ankrd1* and *Nppb*, stress genes that we had already found to be up-regulated in CMs by snRNA-seq at the same time point (*Figures 3E* and *4B* and Supplementary material online, *Table S4*).

DNA motif analysis of sequences captured by ATAC-seq revealed the most enriched motifs after rmIL11 treatment were targets for FOSL2 and JUNB transcription factors (*Figure 4D* and Supplementary material online, *Table S6*). These transcription factors belong to the activator protein-1 (AP-1) transcription factor family, which is important for CM stress responses, cardiac inflammation and fibrosis.^31,32^ Notably, the STAT3 binding motif was also highly enriched.

We revisited our bulk RNA-seq data to examine the expression of the AP-1 transcription factor family transcripts after rmIL11 injection. This revealed that almost all of the AP-1 family transcripts are up-regulated in the heart after rmIL11 injection (*Figure 4E*). We then queried the snRNA-seq data and observed that *Fosl2*, *Junb*, *Atf6*, *Jun*, *Atf3*, and *Mafg* are all significantly differentially expressed in CMs following rmIL11 injection (*Figure 4E* and Supplementary material online, *Table S4*).

### 3.6 Viral-mediated CM-specific deletion of *Il11ra1*

**Figure 5:**
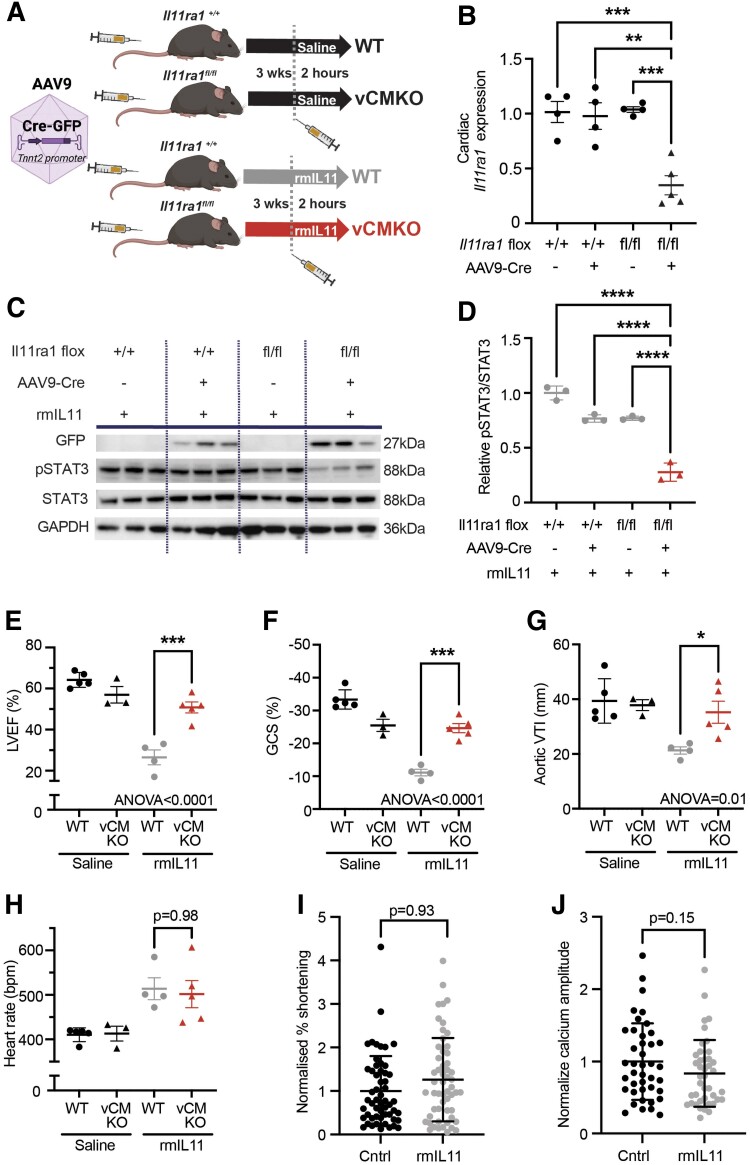
Viral-mediated *Il11ra1* deletion in adult cardiomyocytes protects against IL11-driven cardiac dysfunction. (*A*) Schematic of experimental design for AAV9 mediated delivery of *Tnnt2* promoter driven Cre-recombinase to male *Il11ra1^fl/fl^* or *Il11ra1^+/+^* mice. (*B*) qPCR of relative myocardial expression of *Il11ra1* in *Il11ra1^+/+^* or *Il11ra1^fl/fl^* injected with AAV9-Cre or vehicle. (*C*) Western blot from myocardial lysate following rmIL11 injection (200 µg/kg) in *Il11ra1^+/+^* or *Il11ra1^fl/fl^* treated with either AAV9-Cre or saline (*n* = 3). The membrane was probed with primary antibodies against GFP, pSTAT3, STAT3, and GAPDH. (*D*) Quantification of relative pSTAT3/STAT3 from (*C*). Echocardiographic assessment of vCMKO mice (▴) injected with rmIL11 (200 µg/kg) or saline were compared to WT mice (●) injected with rmIL11 (200 µg/kg) or saline. (*E*) Left ventricular ejection fraction, (*F*) global circumferential strain, (*G*) velocity time integral at the aortic arch, and (*H*) heart rate were measured 2 h after treatment (*n* = 3–5). (*I*) Contractility and (*J*) peak calcium amplitude in CMs isolated from vCMKO mice and treated for 2 h *in vitro* with rmIL11 containing media (10 ng/mL) or normal media. Statistics: one-way ANOVA with Sidak’s multiple comparisons testing. Significance denoted as *P < 0.05, **P < 0.01, ***P < 0.001, ****P < 0.0001. CM data: two-level hierarchical clustering.

Given that profound transcriptional changes occur across multiple cell types in the myocardium, we sought to isolate the effects of IL11 on the CM and test whether the acutely negative inotropic effects of IL11 and CM stress signature are specifically mediated via IL11 activity in CMs. We proceeded to conditionally delete *Il11ra1* in CMs in the adult mouse using an AAV9 vector to express *Tnnt2*-dependent *Cre*-recombinase in CMs of *Il11ra1* floxed mice, which effectively removed the floxed exons to generate mice with viral-mediated deletion of *Il11ra1* in CMs (vCMKO mice) (*Figure 5A* and *B*). Effective transfection in the myocardium was confirmed by immunoblotting for GFP that is co-expressed with the *Cre*-recombinase (*Figure 5C*). Notably, vCMKO mice had diminished myocardial p-STAT3 following injection of rmIL11, confirming IL11 activation of JAK/STAT3 in CMs (*Figure 5C* and *D*).

As compared to mice injected with saline, WT mice injected with rmIL11 had reduced LVEF (WT + rmIL11: 26.5% ± 3.6), whereas vCMKO injected with rmIL11 had a mean LVEF (vCMKO + rmIL11: 50.8% ± 2.7) that was indistinguishable from saline-injected controls (WT + saline: 64.2% ± 1.6; vCMKO + saline: 57.0%±4.0, *n* = 3–5 per group) (*Figure 5E*). Similar changes were seen in GCS (WT + saline: −33.4% ± 1.3; vCMKO + saline: −25.5% ± 1.9; vCMKO + rmIL11: −24.6% ± 1.4; WT + rmIL11: −11.1% ± 1.0, *P* < 0.0001) and VTI in the aortic arch (WT + saline: 37.8 mm ± 2.0; vCMKO + saline: 37.8 mm ± 1.9; vCMKO + rmIL11: 35.2 mm ± 4.03; WT + rmIL11: 21.3 mm ± 1.31, *P* < 0.0371) (*Figure 5F* and *G*). Interestingly, this experimental model still developed tachycardia following IL11 treatment, as seen in WT mice (*Figure 5H*).

We performed experiments in CMs isolated from adult vCMKO mice. Unlike CMs isolated from WT animals (*Figure 1J* and *K*), CM from vCMKO mice did not have a reduction in cell shortening in response to stimulation with rmIL11, as compared to unstimulated cells. Similarly, peak calcium concentration was not blunted by rmIL11 in vCMKO CMs (*Figure 5I* and *J*). As such, IL11 effects in CMs are dependent on *Il11ra1* expression in CMs.

### 3.7 Germline deletion of *Il11ra1* in cardiomyocytes

**Figure 6:**
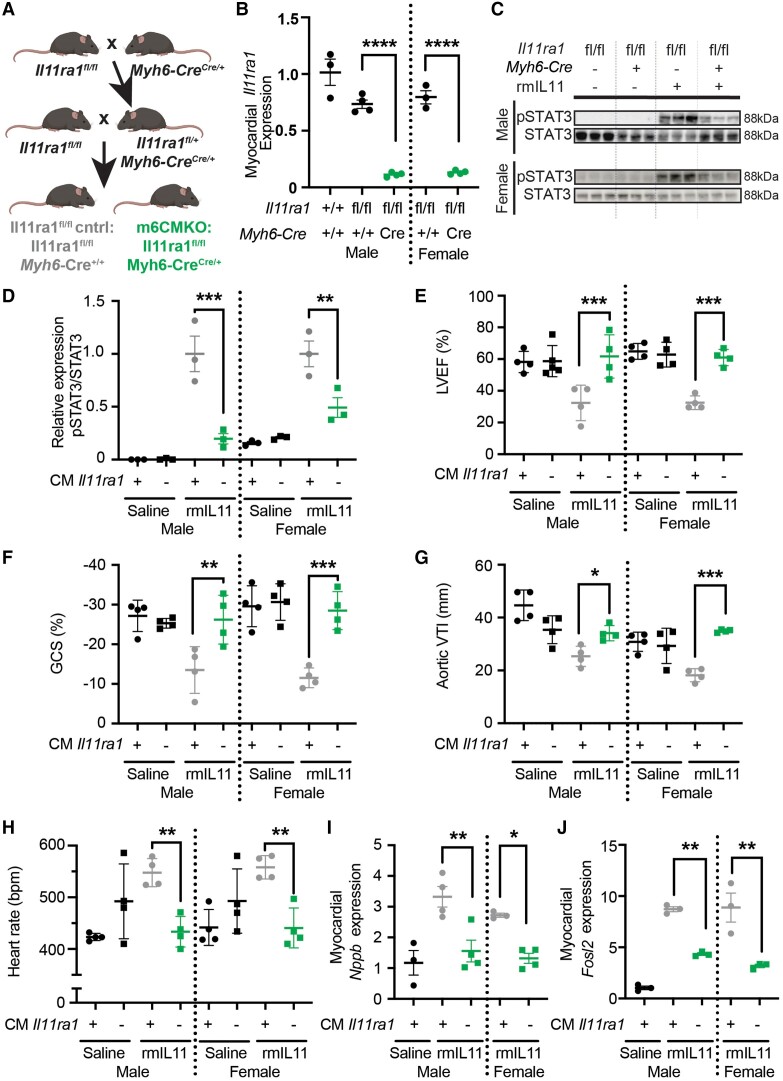
Germline deletion of *Il11ra1* in cardiomyocytes prevents IL11-induced cardiac toxicities. (*A*) Breeding strategy to generate m6CMKO mice and litter-mate *Il11ra1^fl/fl^* controls. (*B*) qPCR of *Il11ra1* gene expression in *Il11ra1^fl/fl^* controls and m6CMKO mice compared to male wild type C57BL/6J controls. (*n* = 4) (*C*) Western blot of phospho-STAT3 and total STAT3 signalling in male and female *Il11ra1^fl/fl^* controls and m6CMKO mice with and without rmIL11 treatment. (*D*) Quantification of relative pSTAT and STAT3 expression. Male and female m6CMKO mice (CM *Il11ra*−) (▪) were treated with saline or rmIL11 and compared to wild type mice (CM *Il11ra1*+) (●) treated with saline or rmIL11 (*n* = 4). (*E*) LVEF, (*F*) GCS, (*G*) VTI in the aortic arch, and (*H*) heart rate were measured 2 h after rmIL11 injection. (*n* = 4). qPCR analysis of relative expression of (*I*) *Nppb* and (*J*) *Fosl2* in the myocardium following rmIL11 treatment of m6CMKO mice and *Il11ra1^fl/fl^* control mice (*n* = 3–4). Statistics: comparison between groups by two-way ANOVA with Sidak’s multiple comparisons. *P*-values denoted as *<0.05, **<0.01, ***<0.001, ****<0.0001.

To strengthen the finding from the initial receptor knockout experiment that negative inotropic effects of IL11 are direct receptor-dependent effects on CMs, we used a complementary, germline deletion methodology. We crossed *Il11ra1* flox mice with *Myh6*-Cre (m6CMKO) mice^33^ (*Figure 6A*) that achieved a more pronounced and consistent knockdown of *Il11ra1* and enabled experiments to be scaled across sexes (*Figure 6B*). As seen in the vCMKO strain, m6CMKO mice of both sexes had reduced p-STAT3 following rmIL11 injection, which further established effective *Il11ra1* locus recombination in this strain and reaffirmed IL11-specific signalling in CMs (*Figure 6C* and *D*).

Having established the m6CMKO strain, we examined the effects of rmIL11 on cardiac function in these mice (Supplementary material online, *Table S7*). When injected with rmIL11, control mice (*Il11ra1^fl/fl^*, *Myh6*-Cre^−/−^) had significantly reduced LVEF whereas the LVEF of m6CMKO mice (*Il11ra1^fl/fl^*, *Myh6*-Cre^+/−^) was similar to that of m6CMKO mice injected with saline (*Figure 6E*). Similarly, following rmIL11 injection, GCS and VTI in the aortic arch were reduced in control mice expressing *Il11ra1* but not in m6CMKO mice (*Figure 6F* and *G*). It was evident that the molecular and cardiovascular phenotypes of m6CMKO mice injected with rmIL11 largely replicated those observed in the vCMKO mice. However, unlike the vCMKO strain, m6CMKO mice were protected against IL11-induced tachycardia (*Figure 6H*).

In molecular studies of myocardial extracts, *Nppb* and *Fosl2*, the most strongly up-regulated CM-specific AP-1 transcript, were up-regulated in *Il11ra^fl/fl^* control mice in response to rmIL11 injection but this was not seen in m6CMKO mice (*Figure 6I* and *J*).

### 3.8 JAK inhibition protects against IL11-induced cardiac dysfunction

**Figure 7:**
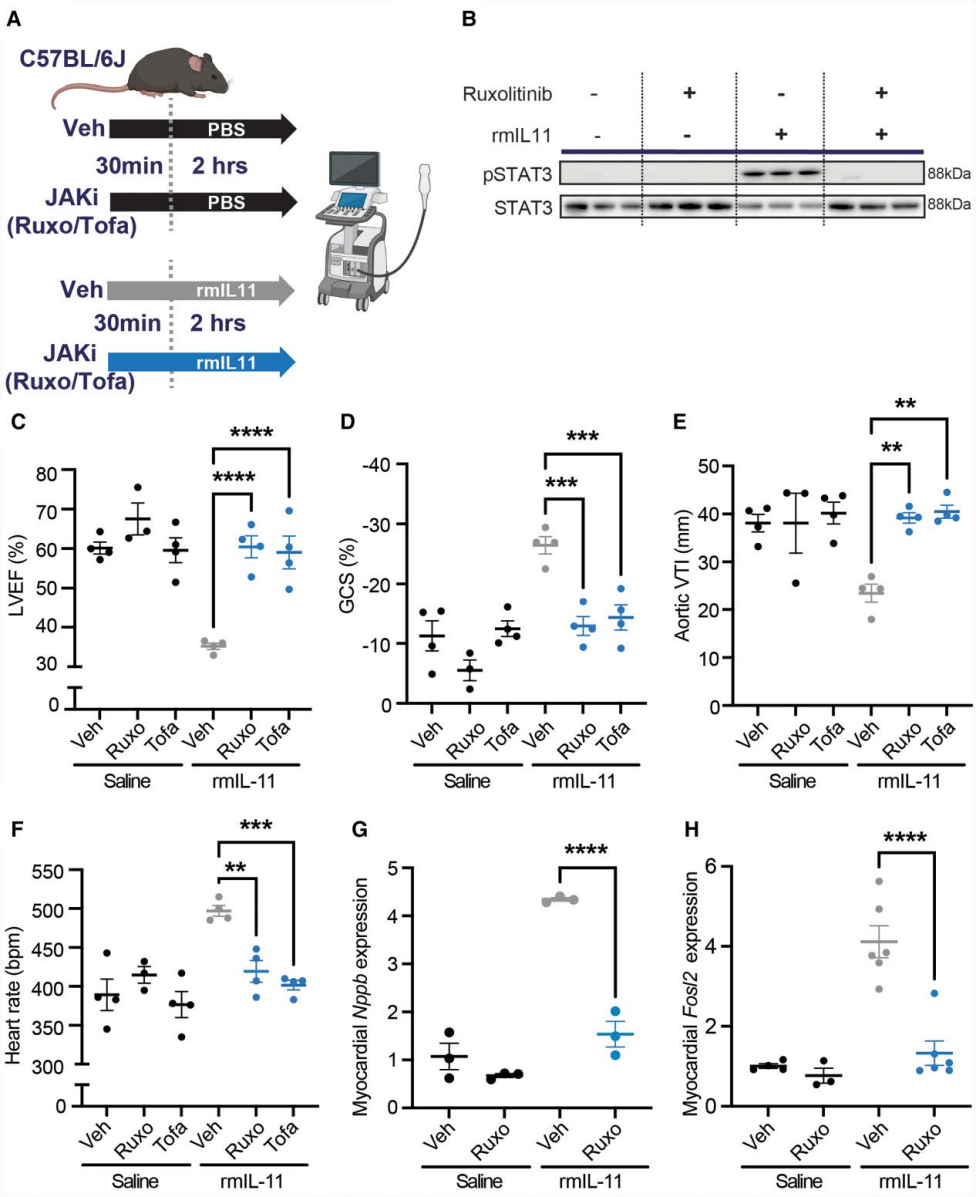
The acute toxic effects of rmIl11 are mediated via JAK/STAT signalling. (*A*) Schematic of the pre-treatment of wild type male C57BL/6J mice with JAKi or vehicle 30 min before administration of rmIL11 or saline. (*B*) Western blot of myocardial lysate from mice 1 h after injection with saline or rmIL11 following pre-treatment with either ruxolitinib (30 mg/kg) (Ruxo) or vehicle (Veh). Membranes have been probed for pSTAT3, STAT3, and GAPDH (*n* = 3). Two hours after treatment, mice had an echocardiogram performed under isoflurane anaesthesia that measured (*C*) left ventricular ejection fraction, (*D*) global circumferential strain, (*E*) VTI in the aortic arch, and (*F*) heart rate (*n* = 4) in mice treated with a combination of vehicle (Veh), ruxolitinib (30 mg/kg) (Ruxo), or tofacitinib (20 mg/kg) (Tofa) and rmIL11 or saline. (*G*) qPCR of *Nppb* and (*H*) *Fosl2* expression in myocardial tissue from combinations of ruxolitinib and rmIL11 treatments (*n* = 3–6). Statistics: comparison between groups by one-way ANOVA with Sidak’s multiple comparisons test. Significance denoted as *P < 0.05, **P < 0.01, ****P < 0.0001.

Canonical IL11 signalling through the IL11RA/gp130/JAK/STAT3 pathway has recently been implicated in the acute pro-inflammatory effects of IL11,^34^ and activation of STAT3 in the heart was immediate and pronounced following IL11 injection (*Figure 1I*). To determine the functional relevance of JAK/STAT3 activation in the heart, we pre-treated mice with ruxolitinib (30 mg/kg), which inhibits JAK1/2 activation, prior to injection of rmIL11 (*Figure 7A*).

We confirmed that administration of ruxolitinib at 30 mg/kg prevented activation of JAK/STAT3 signalling by immunoblotting (*Figure 7B*). Having established the efficacy of ruxolitinib, we studied its effect on cardiac physiology in 8-week-old wild type male C57BL/6J mice injected with rmIL11. Ruxolitinib alone had no effect on LV function (*Figure 7C*). Following injection of rmIL11, and as compared to buffer injected controls, mice pre-treated with ruxolitinib had better LVEF (Ruxo + rmIL11: 60.5% ± 2.79; Veh + rmIL11: 35.2% ± 0.79, *P* = 0.0005, *n* = 4), GCS (Ruxo + rmIL11: −27.1% ± 1.56; Veh + rmIL11: −13.6% ± 1.44, *P* = 0.0009, *n* = 4), and aortic VTI (Ruxo + rmIL11: 39.2 mm ± 10.9; Veh + rmIL11: 23.4 mm ± 1.92, *P* = 0.0001, *n* = 4) (*Figure 7C–E*). Ruxolitinib pre-treatment also prevented rmIL11-induced tachycardia (Ruxo + rmIL11: 497 ± 6.8; Veh + rmIL11: 419 ± 14.1, *P* = 0.0008, *n* = 4) (*Figure 7F*). As seen with m6CMKO, JAK inhibition prevented stress-associated transcriptional changes in the heart of *Nppb* and *Fosl2* (*Figure 7G* and *H*).

To exclude off-target effects and to replicate findings, the study was repeated with a second JAK inhibitor (tofacitinib, 20 mg/kg). As seen with ruxolitinib, pre-treatment with tofacitinib protected against the varied deleterious effects of IL11 on cardiac function compared to vehicle-treated controls: LVEF (Tofa + rmIL11: 59.0% ± 4.2, *P* = 0.0007), GCS (Tofa + rmIL11: −25.7% ± 2.1, *P* = 0.002), VTI in the aortic arch (Tofa + rmIL11: 40.5 mm ±1.36, *P* < 0.0001), and tachycardia (Tofa + rmIL11: 401 bpm ± 6.23, *P* = 0.0002) (*Figure 7C–E*).

## 4. Discussion

In some healthcare systems, rhIL11 is used routinely to increase platelet counts in patients with thrombocytopenia but this can cause serious cardiac complications that are unexplained and until now dismissed as non-specific. RhIL11 has also been trialled in a different context, as a cytoprotective agent, in patients across a range of other medical conditions (e.g. colitis, myocardial infarction, arthritis, and cirrhosis) (*Table 1* and Supplementary material online, *Table S1*) as IL11 was previously thought to be anti-inflammatory and anti-fibrotic.^16^ As such, many thousands of patients have received, and continue to receive, rhIL11 in clinical trials and as part of routine medical care. Long-acting formulations of rhIL11 have recently been devised and new clinical trials of rhIL11 are proposed.^5^

While unexplained, the cardiac side effects of rhIL11 have long been recognized and a small clinical trial was initiated in 2009 to determine if rhIL11 (50 µg/kg) affected cardiac conduction (NCT00886743). This trial was terminated prematurely at the request of the sponsor, and no formal conclusions were made. Other studies looking at the effects of injection of human IL11 to adult rats showed no effects on cardiac phenotypes and studies of human atrial myocytes were similarly negative.^35,36^ We suggest that, for these reasons, the severe cardiac side effects of rhIL11 therapy have been explained away as indirect, non-specific effects and thus sidelined.^36^

The findings of this study redress the earlier literature on IL11 activity in the heart where it was believed to be anti-fibrotic,^14^ which appears inaccurate,^30^ and that it was cytoprotective in CMs,^13–15^ which we challenge here.

We found that injection of species-matched rmIL11 to mice caused acute and dose-dependent LV impairment that was mediated via IL11’s action in IL11RA1 expressing CMs. In response to rmIL11 exposure, CMs develop a ‘stressed’ phenotype with genes including *Ankrd1*, *Ankrd23*, *Xirp2*, and *Nppb*. This mirrors transcriptional changes in human CMs from the border zone of myocardial infarcts.^37^ In these studies, using pseudotime analysis, ‘prestressed’ CMs expressed *ANKRD1* and the subsequent emergence of AP-1 transcription factors such as *ATF3* and up-regulation of their target genes herald the transition from prestressed to stressed state accompanied by expression of *NPPB*.

Powerful enrichment of the AP-1 family of transcription factors following rmIL11 injection was seen in bulk RNA-seq, snRNA-seq, and ATAC-seq and was dependent upon the CM IL11 receptor and JAK signalling. Up-regulation of this family of transcription factors was unexpected and likely has detrimental effects in the mouse heart.^31,38^ AP-1 family activation is not immediately downstream of IL11:IL11RA:gp130 signalling and thus, the early IL11-stimulated activation of JAK/STAT3 appears to up-regulate AP-1 transcription factors in the CM, priming the cell to respond to stress signals. In the injured zebrafish heart, AP-1 contributes to sarcomere disassembly and regeneration,^39^ which is IL11-dependent,^40^ providing an evolutionary context for IL11-mediated effects in the heart.^41^ Similarly, the increase in CM ribosomal proteins seen in the single nuclei RNA sequencing data may be priming the cell for this process; however, in the absence of regenerative potential of these cells, this does not proceed.

Our use of two mouse models of CM-specific *Il11ra1* deletion shows and replicates that the effects of rmIL11 on cardiac function are via direct cardiotoxic effects on CMs and are not explained by changes in circulating volume, as has previously been suggested^36^ or secondary effects on other organ systems. The models used in this study involved the administration of a single dose of rmIL11; however, in clinical practice, courses of therapy can involve daily infusions of rhIL11 for up to 21 days between chemotherapy cycles that are likely to compound the effect on the heart, specifically on fibrotic pathologies that are slower to establish.^30^

The mechanisms underlying the cardiac dysfunction, while localized to CMs, are likely multifactorial and a number of candidates may be considered. *Rrad* is one of the most strongly up-regulated transcripts at 1 and 3 h (Supplementary material online, *Figure S6A*). The *Rrad* protein product, RAD-GTPase, is a well-characterized L-type calcium channel inhibitor,^42,43^ and its up-regulation has been described in human myocardial infarction under the control of the AP-1 family transcription factor *ATF3*.^37^ In our studies, *Rrad* expression is dependent on the CM IL11 receptor, as vCMKO, m6CMKO, and JAKi prevent the IL11-induced up-regulation of this transcript (Supplementary material online, *Figure S6B*–*E*). Similarly, increased expression of acute phase alarmins S100A8 and S100A9 is seen 1 and 3 h after rmIL11 injection (Supplementary material online, *Figure S6F* and *G*). These genes have both been previously implicated in impairment of CM calcium flux and myocardial depression in the setting of acute inflammation.^44^ These candidates, and others, may be considered for investigation in follow-on studies.

There are several limitations to our study. The discrepancy between the tachycardia seen in vCMKO but not m6CMKO mice was not explored. Mice developed a marked tachycardia in response to rmIL11 therapy that can cause changes in ventricular function. It was not possible to isolate the effect of IL11 on ventricular function without the concurrent tachycardia; however, LVEF will typically increase in response to elevated heart rates. In some cases where tachycardia is profound, end-diastolic volume and therefore stroke volume can be decreased due to the shortened filling time. However, in our study, the end-diastolic volume increased after rmIL11 administration (*Table 2*) suggesting that tachycardia was unlikely to play a major role in the change in cardiac output and studies in unloaded and paced CMs *ex vivo* provide orthogonal evidence of IL11 pathobiology on myocyte contraction and relaxation. It is known that IL11 is produced endogenously in the heart in mice following transverse aortic constriction and angiotensin II infusion^45^ and in humans with atrial fibrillation^46^ and heart failure.^47^ However, whether endogenous IL11 is toxic to CMs and negatively inotropic in heart failure syndromes is not known and we cannot extrapolate from the data seen with acute, high dose injection of recombinant protein. The cardiac side effects associated with IL11 include arrhythmias (notably atrial fibrillation and flutter) that we did not study here.

In conclusion, we show for the first time that injection of IL11 at doses equivalent to those used in clinical practice causes IL11RA-dependent, CM-specific toxicities, and acute heart failure. These data likely explain the serious cardiac side effects that occur with rhIL11 therapy. Previous studies in human and non-human primates have shown an association between IL11 administration and heart failure symptoms, myocardial hypertrophy, and elevation in natriuretic peptides.^5,47^ These associations combined with our data mechanistic data strongly question the ongoing use of rhIL11, and its further development, in patients with thrombocytopenia while identifying novel toxic effects of IL11 in the CM compartment of the heart.

**Translational perspective**

Injection of IL11 into mice causes acute and dose-dependent left ventricular impairment by activation of JAK/STAT3 signalling in cardiomyocytes that induces cell stress, inflammation, and impaired calcium handling. These data identify, for the first time, that IL11 is directly toxic in cardiomyocytes, overturning the earlier literature that suggested the opposite.

Recombinant human IL11 (rhIL11) is used as a drug to increase platelets in patients with thrombocytopenia but this has severe and unexplained cardiac side effects that were previously believed sporadic and non-specific. These findings have translational implications as in combination with previously described side effects of rhIL11 in clinical practice they question the continued use of rhIL11 in patients around the world.

## Supplementary Material

cvae224_Supplementary_Data

## Data Availability

The data underlying this article are available in the article and in its online supplementary material. Raw RNA-seq data and gene-level counts have been uploaded onto the NCBI Gene Expression Omnibus database with accession number (GSE240804). All single nuclei sequence data generated and analysed in this study have been deposited in the European Nucleotide Archive (ENA) at EMBL-EBI under accession number PRJEB67301 (https://www.ebi.ac.uk/ena/browser/view/PRJEB67301).
