# Supplementary material for: Interleukin 11 therapy causes acute left ventricular dysfunction
Source: Cardiovasc Res. 2024 Oct 9;120(17):2220–35. doi: 10.1093/cvr/cvae224 (PMC11687394; doi:10.1093/cvr/cvae224)
Supplement: cvae224_Supplementary_Data [file cvae224_supplementary_data.zip › IL11 effects of cardiomyocytes_uncropped blots.pdf]

Figure 1I

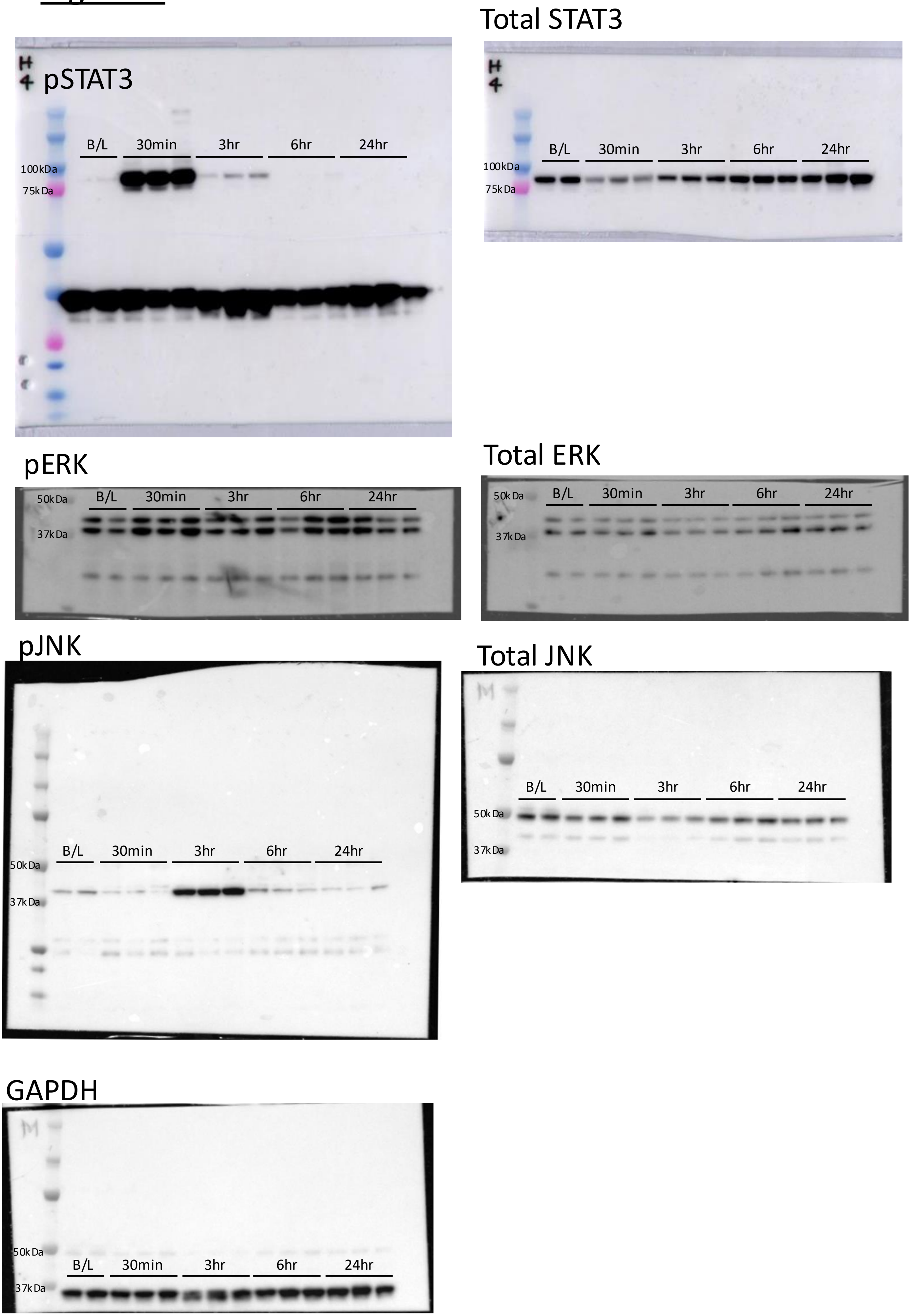
pSTAT3
B/L
30min
3hr
6hr
24hr
100kDa
75kDa
Total STAT3
B/L
30min
3hr
6hr
24hr
100kDa
75kDa
pERK
50kDa
37kDa
B/L
30min
3hr
6hr
24hr
Total ERK
50kDa
37kDa
B/L
30min
3hr
6hr
24hr
pJNK
B/L
30min
3hr
6hr
24hr
50kDa
37kDa
Total JNK
B/L
30min
3hr
6hr
24hr
50kDa
37kDa
GAPDH
50kDa
B/L
30min
3hr
6hr
24hr
37kDa

Figure 5C

GFP

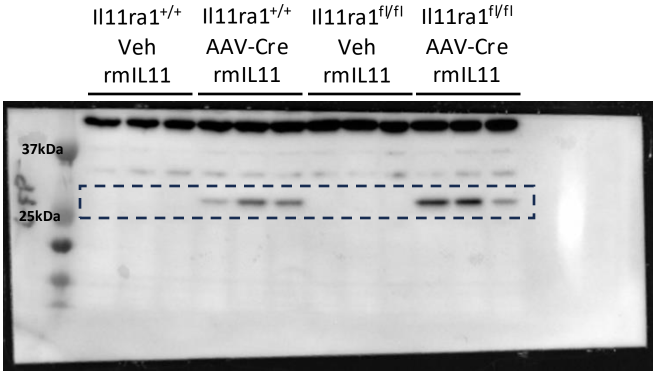

pSTAT3

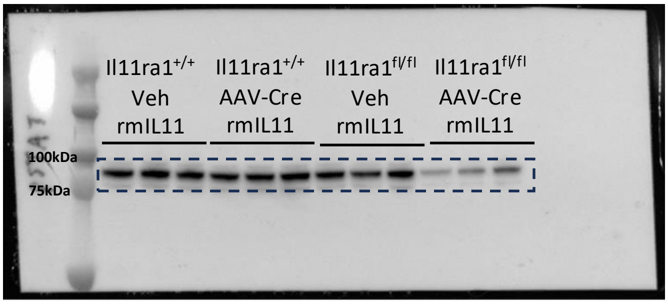

Total STAT3

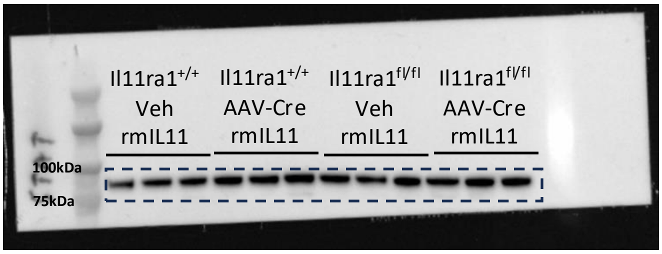

GAPDH

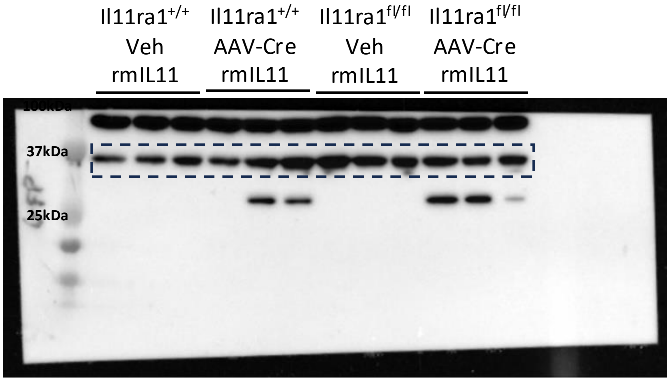

Figure 6C

Males

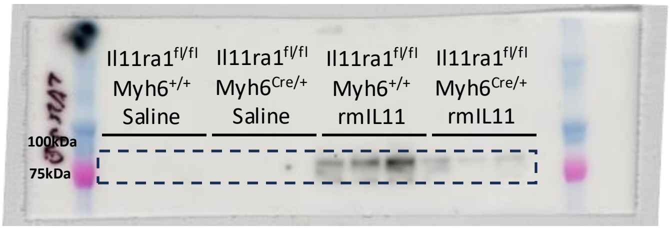

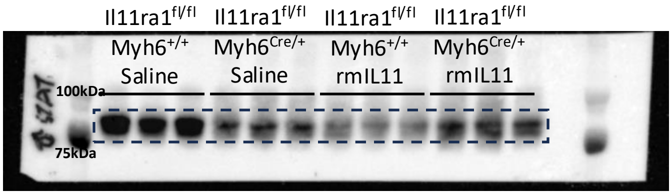

Females

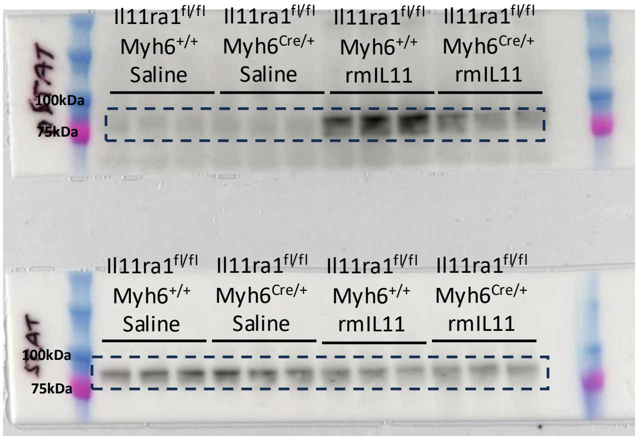

Figure 7B

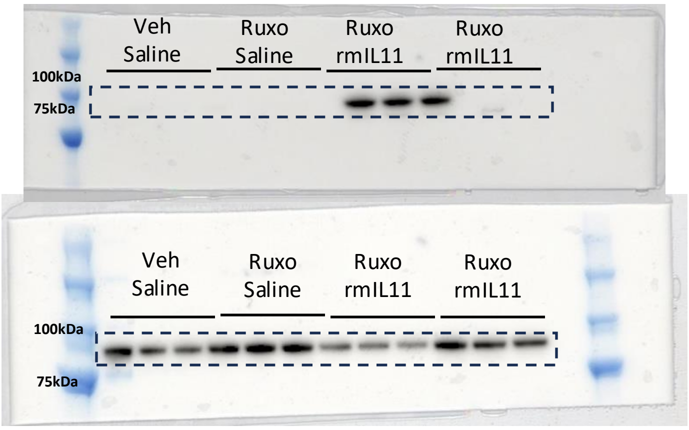

Veh
Saline
Ruxo
Saline
Ruxo
rmIL11
Ruxo
rmIL11
100kDa
75kDa
Veh
Saline
Ruxo
Saline
Ruxo
rmIL11
Ruxo
rmIL11
100kDa
75kDa
